# Oxygen Use in Neonatal Care: A Two-edged Sword

**DOI:** 10.3389/fped.2016.00143

**Published:** 2017-01-09

**Authors:** Serafina Perrone, Carlotta Bracciali, Nicola Di Virgilio, Giuseppe Buonocore

**Affiliations:** ^1^Department of Molecular and Developmental Medicine, General Hospital “Santa Maria alle Scotte”, University of Siena, Siena, Italy

**Keywords:** oxygen, reactive oxygen species, free iron, mitochondria, oxidative stress, newborn infants

## Abstract

In the neonatal period, the clinical use of oxygen should be taken into consideration for its beneficial and toxicity effects. Oxygen toxicity is due to the development of reactive oxygen species (ROS) such as OH^•^ that is one of the strongest oxidants in nature. Of note, generation of ROS is a normal occurrence in human and it is involved in a myriad of physiological reactions. Anyway an imbalance between production of oxidant species and antioxidant defenses, called oxidative stress, could affect various aspect of organisms’ physiology and it could determine pathological consequences to living beings. Neonatal oxidative stress is essentially due to decreased antioxidants, increased ROS, or both. Studies have demonstrated that antioxidant capacity is lower in preterm newborns than term babies. This well-known deficiency of antioxidant factors is only a piece of a cohort of factors, which can be involved in the neonatal oxidative stress and the increased production of ROS may be a main factor. Mechanisms of ROS generation are: mitochondrial respiratory chain, free iron and Fenton reaction, inflammation, hypoxia and/or ischemia, reperfusion, and hyperoxia. Oxidative stress following hyperoxia has been recognized to be responsible for lung, central nervous system, retina, red blood cell injuries, and possibly generalized tissue damage. When supplemental oxygen is needed for care, it would be prudent to avoid changes and fluctuations in SpO_2_. The definition of the safest level of oxygen saturations in the neonate remains an area of active research. Currently, on the basis of the published evidences, the most suitable approach would be to set alarm limits between 90 and 95%. It should allow to avoid SpO_2_ values associated with potential hypoxia and/or hyperoxia. Although the usefulness of antioxidant protection in the neonatal period is still under investigation, the risk of tissue damage due to oxidative stress in perinatal period should not be underestimated.

## Introduction

Oxygen is essential for aerobic life, but it can be considered a double-edged sword in perinatal period having both positive biological benefits and toxicity effects (1–3). Oxygen toxicity is due to the development of reactive oxygen species (ROS), such as the superoxide anion $\left({\text{O}}_{2}^{-}\right)$, hydrogen peroxide (H_2_O_2_), lipid peroxide (LOOH), peroxyl radicals (RO^•^), electron delocalized phenoxyl radical (C_6_H_50_), nitric oxide (NO), and the hydroxyl radical (OH^•^) (4). OH^•^ is a potent oxidant in biological fluids and may damage tissues, through reaction with lipids, proteins, DNA, amino acids, and several other molecules (5).

Despite their well-known harmful effects on cells, ROS reactions are also implicated in a myriad of physiological reactions, cell fate decisions, and signal transduction pathways. They have a key role in various cellular processes such as energy metabolism, gene expression, protein import, or folding, and they are produced in response to a variety of ligands including growth factors, cytokines, and G protein coupled receptors (6, 7).

Shift in redox potential may favor beneficial or detrimental consequences according to various factors. Both high levels of ROS and excessive low levels of ROS can alter the balance between pro-oxidant and antioxidant elements, which is essential for biologic processes (8). An imbalance between oxidants and antioxidants is called oxidative stress and that is a potential cause of damage (9). If oxidative stress is mild, cell defenses may increase by a mechanism, which generally involves enhanced gene expression of ROS scavenging activities (10). On the other hand, severe oxidative stress is generally followed by lipid peroxidation that alters membrane structure, disrupts membrane permeability properties, and alters cellular components. Abnormalities in cell membrane proteins due to high levels of ROS can also induce functional consequences including, for instance, alteration in recognition of cells in the immune response (11), apoptosis, and/or necrosis (12).

Oxidative stress in the newborn may result from decreased antioxidants, increased ROS, or both. Antioxidant capacity is lower in the newborn and particularly the premature infant in comparison to term newborn (13, 14). The level and activity of the most-relevant antioxidant enzymes, such as superoxide dismutase (SOD), catalase (CAT), and glutathione peroxidase (GPX), change dynamically during development and mature in the last weeks of gestation, preparing the fetus for lung respiration (15–17). Non-enzymatic antioxidant factors, such as α-tocopherol and reduced glutathione (GSH), are low in the fetus and newborns (18, 19). Therefore, premature infants are especially prone to oxidant injury, being unprepared for hyperoxic challenge of extrauterine life. It is demonstrated that a 30 min exposure to 100% O_2_ at birth can cause a significant increase in lipid peroxidation in live newborn sheep (14).

The deficiency of antioxidant factors, that is characteristic of the neonate, is only a piece of a cohort of factors which can be involved in the neonatal oxidative stress and the increased production of ROS may be an additional factor. Some studies demonstrated that, in the immature lung of preterm newborn, the main sources of ROS could be ischemia, reperfusion, phagocytosis, and hyperoxia (20–22).

The various pathways of ROS generation should be considered and it is necessary to take into account the complexity of redox equilibrium and, therefore, correctly interpreting the origin of oxygen toxicity in newborn.

This review focuses on the mechanisms of ROS production and ROS-induced toxic effects following oxygen administration in newborns, by considering both short- and long-term consequences of oxidative stress exposure. Furthermore, it deals with the recent research on the definition of the safest level of oxygen saturations in the neonatal period and the state of knowledge on oxygen use in clinical practice.

## ROS: Biochemistry and Biology

Molecular oxygen (O_2_) has two unpaired electrons in separate orbitals in its outer electron shell. This chemical structure enhances ROS generation (23).

In general, the principal endogenous sources of ROS in human and, in particular, in newborn are mitochondrial metabolism, increased free circulating transition metals, inflammation through NADPH oxidase (NOX) reactions, hypoxia–reoxygenation (through hypoxanthine–xanthine oxidase reaction), hyperoxia, and paradoxically hypoxia (24–29). These mechanisms will be discussed in turn.

### Mitochondrial Respiratory Chain

Mitochondrial respiratory chain is the main source of ROS. Mitochondria play a key role for the ATP production in eukaryotic cells. The sequence of events involved in oxidative phosphorylation, which takes place in mitochondria, leads to ATP formation as a result of the transfer of electrons from NADH to O_2_, by a series of electron carriers (30).

Initially, electron donors can convert O_2_ to ${\text{O}}_{2}^{-}$. Dismutation of ${\text{O}}_{2}^{-}$ by superoxide dismutase (SOD) produces H_2_O_2_ that in turn may be fully reduced to water (H_2_O) by glutathione peroxidase (GSH-Px) and catalase (CAT) or, alternatively, partially reduced to the OH^•^ in the Fenton–Haber Weiss reaction, catalyzed by reduced transition metals, particularly iron, but also copper and zinc (24). Under physiologic conditions, approximately 98% of O_2_, undergoes a complete reduction to form H_2_O_2_, whereas 2% of electrons will leak, causing a partial reduction of the oxygen and producing ROS. ROS generation by mitochondria is mainly dependent on complexes I and III and is highly dependent on metabolic conditions and on the intra-mitochondrial balance between oxidative and antioxidative factors (6, 31).

### Free Iron and Fenton Reaction

Iron could be considered a two-edged sword for living organisms and, in particular, for newborns (32). It is an essential transition metal for the proper growth and normal neurologic development but it is toxic when unbound. Under conditions of body iron overload, plasma transferrin becomes fully loaded with iron, and chelatable forms of iron escape sequestration in biological systems. They become available to react with reduced oxygen, finally generating the toxic OH^•^ (33). Non-protein bound iron easily enters in the Fenton–Haber Weiss reaction: H_2_O_2_ generated by dismutation of ${\text{O}}_{2}^{-}$ can break down, in presence of ferrous ion, to produce the most damaging of the oxygen free radicals, the OH^•^ (25), and to form ferric ion (34).

### Inflammation

Respiratory burst of phagocytic cells by NOX is a known source of ROS production in mammalian cells (12). While the most relevant generation of ROS by NOX occurs in phagocytes after activation upon exposure microbes, microbial products, or inflammatory mediators (8), ROS are produced *via* NOX in a variety of cell type and in response to normal physiological signals such as insulin, angiotensin II, growth factors, and various classes of receptors, such as formylpeptide receptors and toll-like receptors (35, 36). Furthermore, NOX-dependent ROS generation has been suggested to trigger adaptive response of a variety of stressors (36). Opsonization and activation of phagocytes are also known to occur as consequences of hypoxia, hypoxanthine–xanthine oxidase reaction, and hypoxia–reoxygenation (37). However, NOX-induced ROS generation can activate the NF-E2 related factor 2 pathway, which increases antioxidant protection during inflammation (38).

### Hypoxia and/or Ischemia

Metabolic conditions and O_2_ levels modify the rate of ROS generation (39). Hypoxia and/or ischemia results in increased electron leakage, and the interaction of various activated signals with residual oxygen produces superoxide. In animal models, several studies have demonstrated that hypoxia increases lipid peroxidation by peroxynitrite production and decreases Na^+^, K^+^-ATPase activity leading to cellular membrane dysfunction. Moreover, hypoxia induces modification of the *N*-methyl-d-aspartate receptor-ion channel complex, leading to increased intracellular Ca^2+^. Intracellular calcium activates several enzymes, such as proteases, potentiating free radical generation and resulting in hypoxic cell injury (40).

Furthermore, during hypoxia, redox signals to and from mitochondria are activated. In particular, the respiratory chain increases ROS production stimulating the signaling pathway to induce hypoxia-induced factor (HIF)-dependent gene expression. HIF-1 is an important protein causing a shift from aerobic to anaerobic metabolism and also reducing mitochondrial oxygen consumption. Thus, it seems that the byproducts of oxidative phosphorylation play a role as signaling molecules, conveying cellular oxygen availability (41).

### Reperfusion

Reperfusion is the second phase of ischemia/reperfusion (I/R) injury, and it is characterized by the generation of ROS when circulation is restored. In this phase, the reestablishment of blood supply to ischemic tissues causes the delivery of blood-borne elements (platelets and leukocytes) that are activated by and release ROS. ROS may induce cell damage and death by interacting with NO, fatty acids, or non-protein bound iron to generate more toxic free radicals such as peroxynitrite, peroxyl radicals, and hydroxyl radicals. Moreover oxygen free radicals facilitate the inflammatory response to reperfusion, by making oxidant-dependent pro-inflammatory mediators (11, 31).

A third stage of I/R injury constitutes the reparative phase. ROS promote angiogenetic growth factors, vascular remodeling, activation of matrix metalloproteinases that contribute to fibrosis, and formation of scar tissue (31).

### Hyperoxia

Hyperoxia could be defined as a state of excess supply of O_2_ in tissues and organs. The inhalation of a high level of oxygen, has been reported to be followed by membrane bound NOX activation, free radical generation, and DNA damage with apoptosis (42). At birth, blood oxygen content and oxygen availability sharply increase to their adult values, eliciting the production of a flood of ROS (43, 44), which may act as signaling molecules in specific metabolic pathways, in response to oxidative stress (45, 46).

In animals, exposed to high oxygen concentration, a modification of nuclear membrane function has been reported as consequence of high nuclear Ca^2+^ influx, activation of Ca^2+^/calmodulin-dependent protein kinase pathway, and CREB protein-mediated apoptotic proteins (47).

Furthermore, hyperoxia is involved in activation of a panel of pro-inflammatory cytokines, including IFNγ and macrophage inflammatory protein 2, that, in turn, could finally develop ROS.

## Oxygen Toxicity

In the clinical settings, ROS generation following hyperoxia has been recognized to be responsible for lung, central nervous system, retina, and red blood cell injuries as well as generalized tissue damage, which can be reported both in the neonatal period and in the adult life.

Focusing on neonatal period, the following paragraphs explain the mechanisms of both the short- and the long-term toxic effects of oxygen administration and hyperoxia on various organs and body systems.

### Short-term Adverse Effects

#### Lung

Hyperoxia is particularly harmful for the lungs and the mechanism of damage is complex. Chronic oxygen toxicity may damage the pulmonary epithelium and inactivate the surfactant, form intra-alveolar edema and interstitial thickening, and later fibrosis, leading to pulmonary atelectasis (48). Lung injury is demonstrated to be caused directly by ROS production in response to hyperoxia and indirectly by ROS due to phagocyte activation and inflammation. The two mechanisms seem to be integrated (49). *In vitro* and *in vivo* exposures to hyperoxia result in downregulation of peroxisome proliferators-activated receptor gamma and in increase transdifferentiation of pulmonary protective lipofibroblasts to myofibroblasts (MYFs) (50, 51). Epithelial cell growth and differentiation is not adequately supported by MYFs. This results in a disturbed alveolarization, characterizing bronchopulmonary dysplasia (BPD) (52). High level of neutrophils, IL-8, and leukotrienes in alveolar fluid of BPD infants clearly support the role of inflammation and ROS in the development of this oxidative damage (53).

#### Retina

The exposure to hyperoxia is also associated with higher risk for severe retinopathy of prematurity (ROP), due to susceptibility of the phospholipid-rich retina to ROS (54).

The peripheral temporal portion of the retina is the last to be vascularized, and it is still immature even at term (55). With exposure to excess oxygen, the developing retinal endothelial cells activate various transcription factors, including HIF-1α and vascular endothelial growth factor, which, in turn, cause both cessation of retinal vessel growth and loss of some existing retinal vessels (56). These mechanisms finally lead to abnormal retinal vascular proliferation and the formation of a ridge, which places traction on the retina and increases the risk of detachment, as seen in ROP (57).

#### Red Blood Cells

Newborn erythrocytes are more prone to damaging effects of oxidative stress and to have higher content of free iron than those of adults. In this context, free radical damage is involved in neonatal hemolytic anemia and particularly of the preterm (58, 59). Furthermore, prolonged exposure to hyperbaric oxygen leads to changes of erythrocytes shape, as a consequence of toxic effects of oxygen on the erythrocyte membranes. In an animal model, various forms of abnormal red blood cells are observed after exposure to high oxygen concentration, and in particular echinocyte shape was dominated (60).

### Long-term Effects

Exposure to hyperoxia at birth can also be related to long-term pathological effects. Oxygen exposure in the neonatal period has been demonstrated to affect lungs of mice by increasing airway reactivity and persistent inflammation with alteration in the innate immunoregulatory pathways that contribute to “poorer resistance” to respiratory viral infections in adulthood (61, 62). Furthermore, the exposure of newborn mice to hyperoxia may lead to long-term cardiac abnormalities, such as left ventricular dysfunctions (63), and neurodevelopmental impairments in adult life, as demonstrated by abnormal behavior, deficits in spatial and recognition memory, small hippocampal dimensions, in the absence of intracranial pathology such as intraventricular hemorrhage or periventricular leukomalacia in the neonatal period (64).

In conclusion, experimental studies and clinical observations demonstrated high susceptibility of the fetus and newborn to oxidative stress. Increased release and decreased detoxification in the newborn appear to be negatively correlated with the gestational age.

## State of Knowledge of Oxygen Use in Neonatal Care

Avoidance of conditions, such as infections, asphyxia, retinal light exposure, iron supplementation, and, in particular, hyperoxia, reduces oxidative stress.

Recent studies, that have been accomplished, have revised the concept of the optimal oxygenation in newborns, children, and adults.

Chow et al. reported the experience of a tertiary neonatal center, where oxygen administration was tritated to optimize neonatal care. To reduce the incidence of ROP, authors recommended to avoid any fluctuation of FiO_2_ and to maintain oxygen saturation within “acceptable” limits, setting up oxygen alarms below 85% and above 93% in newborns <32 weeks of gestation (65). Tin and Gupta compared two populations of high risk newborns kept at O_2_ saturations of 88–98% and 70–90%. They found a decrease of incidence of ROP in the group treated with lower O_2_ saturation without any differences in mortality and morbidity (66). Neonatal outcomes showed that newborns treated with higher level of oxygen had more cognitive disabilities than those treated with lower oxygen, after 10 years (66). A report from the Oxford Vermont Network, in extremely low birth newborns, demonstrates less chronic lung disease and ROP incidence in babies with a target oxygen saturation of <95% than those with oxygen saturation more than 95% (67).

The first two randomized controlled trials (RCT), performed to answer the question of what is the range of optimal saturation by pulse oximetry in preterm infants receiving supplemental oxygen, were the Supplemental Therapeutic Oxygen for Prethreshold Retinopathy Of Prematurity (STOP-ROP) study (68) and the Benefits of Oxygen Saturation Targeting (BOOST) I (69). In the first, the authors concluded that there was no significant difference in the rate of progression to threshold ROP in group of newborns cared for with lower O_2_ saturation range (89–94%) vs. the higher group (96–99%). But, as secondary outcome, they showed an increased incidence of chronic lung disease and a longer duration of hospitalization, both in the higher group. In the BOOST I, no differences were found in the primary outcomes, defined as growth and neurodevelopmental measures at a corrected age of 12 months, in the two groups (91–94% vs. 95–98%). In the high-saturation group (babies kept at 95–98% of O_2_ saturation), the newborns required oxygen for a longer period, had a higher dependence on oxygen at 36 weeks of postmenstrual age and need for home oxygen therapy with higher frequency, despite babies of low-saturation group, kept at 91–94% of O_2_ saturation.

More recently, five large multicenter, masked, RCT were conducted with a similar design and outcome measures to collect data from 5,000 preterm newborns with less than 28 weeks postmenstrual age; they were the Surfactant Positive Pressure and Pulse Oximetry Randomized Trial (SUPPORT) (70), the BOOST II United Kingdom, Australia, and New Zealand (71), and the Canadian Oxygen Trial (COT) (72). Thanks to these data, it was possible to conduct a prospective meta-analysis, NeOProM (73) study, with a primary outcome defined as a composite of death and disability at 18–24 months of corrected age.

The three studies were performed with the same target ranges of oxygen saturation in the two groups: 85–89% in the lower group vs. 91–95% in the higher group.

In the SUPPORT, the primary outcome was a composite of severe ROP, death before discharge from the hospital, or both. The study showed no significant differences in the primary outcome, but the use of a lower range of oxygen saturation results in a decrease of occurrence of severe ROP and an increase of death before the discharge. The SUPPORT was conducted by pulse oximetry systems, with an older software algorithm, despite the other two trials. In the BOOST II and COT, the software algorithm of the oximetry systems changed about at the midpoint of the studies.

The data from the BOOST II showed that a restrictive use of oxygen, with target range of saturation below 90%, is associated with a higher risk of death and necrotizing enterocolitis despite of a reduction of incidence of ROP, significantly increased in the higher group saturation.

The COT study, with a primary outcome defined as death before 18 months of corrected age or survival with one or more disability, do not showed significant differences in the mortality or other outcome, but only a reduction of duration of O_2_ therapy.

Based on these five RCT, the 2013 European Consensus Guidelines on the Management of Neonatal Respiratory Distress Syndrome in Preterm Infants suggested that SpO_2_ should be targeted at 90–95%, in infants with gestational age <28 weeks until 36 weeks (74).

However, there are more unanswered questions and the optimal oxygen saturation range for low birth weight preterm infants remains elusive.

This is mainly due to the several different clinical conditions of preterm newborns. Some authors indicate that 50 and 70 mmHg (75) is the optimal oxygen tension, but it is noteworthy that pulse oximetry ability remains controversial. Oxygen saturation of more than 90% should be carefully considered because to be found related with an arterial oxygen tension of more than 80 mmHg (76).

In clinical practice, the continuous monitoring of oxygen saturation is mandatory to titrate oxygen therapy as better as possible and the routine use of pulse oximetry systems can be considered a very useful approach for the neonatologists, in order to reach this goal. However, the optimal target range for oxygen saturation in the sick newborns and, above all, in the extremely preterm babies is not clear.

The challenge for the clinicians is reaching a balance in the oxygen administration, to avoid the damage and negative outcomes, associated with either hyperoxemia or hypoxemia.

Based on all the actually available evidence and considering the lack of evidence about the influence of many factors such as transfusional status, different gestational ages and underlying diseases, the most careful approach is to avoid both hypoxia and hyperoxia in infants requiring oxygen supplementation. In order to maintain an intended optimal range of SpO_2_ 90–95%, it has been suggested to set the acoustic signals at 91 and 96%, with a delay time not longer than 20 s (77). It is essential to control the low limit as well as the upper limit to prevent excessive fluctuations of oxygen saturation (78, 79).

## Conclusion

Hyperoxia and hypoxia are deeply involved in the development of several neonatal diseases, and the mechanisms are complex and not yet fully understood. However, evidences suggest that both the generation of oxidant species (i.e., free radicals and ROS) and the deficiency of antioxidants may play a role. Hyperoxia and inflammation as well as the episode of hypoxia–reoxygenation and free iron appear to be sources of increased ROS release, which may cause tissue injury either by direct effect or as consequences of endothelium dysfunction and gene alteration, particularly in preterm newborns.

Understanding the effects of O_2_ administration is important for the management of oxygen therapy in newborns, in order to prevent inadvertent cellular and tissue damage caused by hyperoxia, in the patients requiring supplemental oxygenation.

## Author Contributions

SP: wrote a draft and supervised the final manuscript; CB: assisted with preparation of manuscript; NV: assisted with preparation of manuscript; GB: conceived the idea and supervised the final manuscript.

## Conflict of Interest Statement

The authors declare that there is no commercial or financial relationship that could be constructed as a potential conflict of interest.
